# Modulation of Endothelial Inflammatory Signature by an Imidazo-Pyrazolyl Urea Derivative in a Dynamic In Vitro Model of Hypertension

**DOI:** 10.3390/ph19071003

**Published:** 2026-06-28

**Authors:** Matteo Lusardi, Caterina Bodio, Chiara Brullo, Gianfranco Parati, Pier Luigi Meroni, Maria Orietta Borghi, Elena Raschi, Laura Calvillo

**Affiliations:** 1 Department of Pharmacy, Section of Medicinal Chemistry, University of Genova, Viale Benedetto XV 3, 16132 Genova, Italy; matteo.lusardi@edu.unige.it (M.L.); chiara.brullo@unige.it (C.B.); 2 Immunorheumatology Research Laboratory, Istituto Auxologico Italiano, IRCCS, Via Zucchi 18, 20095 Milan, Italy; c.bodio@auxologico.it (C.B.); or p.meroni@auxologico.it (P.L.M.); or o.borghi@auxologico.it (M.O.B.); raschi@auxologico.it (E.R.); 3 Department of Cardiology, Istituto Auxologico Italiano, IRCCS, Via Magnasco 2, 20149 Milan, Italy; gianfranco.parati@unimib.it; 4 Department of Medicine and Surgery, University of Milano-Bicocca, Piazza dell’Ateneo Nuovo 1, 20126 Milan, Italy; 5 Dipartimento di Scienze Cliniche e di Comunità, Dipartimento di Eccellenza 2023–2027, University of Milan, Via Festa del Perdono 7, 20122 Milan, Italy

**Keywords:** imidazo-pyrazoles, hypertension, inflammation, endothelial cells, bioreactor, AngiotensinII, Live-Pa System

## Abstract

**Background/Objectives**: Hypertension is a multifactorial condition in which inflammation plays a pivotal role. Current preclinical models fail to fully capture the complexity of the underlying mechanisms, limiting therapeutic discovery, while compounds targeting endothelial inflammatory pathways may offer promising alternatives. **Methods**: An advanced in vitro model of hypertension, consisting of a bioreactor for endothelial cell culture coupled with a peristaltic pump to mimic blood circulation, and a pressure modulator as mechanical stimulus (Live-Pa System) to reproduce hemodynamic stimuli, was employed to investigate the effects of the imidazo-pyrazolyl urea (IPU) **3l**, known for its chemotaxis inhibition properties. The effects of **3l** were investigated on hypertension-related inflammatory/vasoconstrictor markers, alone or in combination with hypertensive stimuli (ANGII and/or Live-Pa). **Results**: IPU **3l** effectively counteracted ANGII-induced inflammation by significantly reducing NF-κB activation across all experimental conditions (static, dynamic, and Live-Pa) and IL-8 secretion under static and dynamic conditions. **Conclusions**: IPU **3l** exhibits a consistent anti-inflammatory profile, primarily through inhibition of ANGII-induced NF-κB activation across all experimental conditions, with additional context-dependent effects on IL-8 secretion. This study introduces a new strategy for drug discovery by distinguishing biochemical from mechanical stress, providing a clearer framework to interpret condition-specific pharmacological responses. Such insights, difficult to obtain with conventional in vitro or in vivo models, are essential for developing more effective cardiovascular therapies.

## 1. Introduction

The drug discovery process is a complex and multidisciplinary pathway involving the identification, optimization, and evaluation of new chemical entities. After hit identification, compounds undergo structural optimization to improve their pharmacological, pharmacokinetic (PK) and pharmacodynamic (PD) properties, leading to selected candidates for preclinical testing in in vitro and in vivo models [1,2,3]. Animal models (e.g., mice, rats, and non-human primates) are used to assess compound efficacy and safety in living organisms, but they present limitations in predicting human responses and raise ethical and economic concerns. Despite preclinical validation, only a small proportion of compounds successfully progress through clinical trials, with approximately 70% passing Phase I, 33% Phase II, and 25–30% Phase III [4,5].

Hypertension, one of the most significant risk factors for cardiovascular disease worldwide, represents a particularly critical area within the challenging landscape of drug discovery, as the identification of novel and more effective therapeutic agents is especially important [6,7]. In fact, although extensive preclinical research has contributed to the development of important therapeutic strategies [8,9], several questions remain unresolved and many hypertensive patients still do not adequately benefit from current treatments. One of the problems is essential hypertension, a condition characterized by elevated blood pressure, apparently without a clear cause, and resistance to anti-hypertensive drugs [10]. A hallmark of hypertension is inflammation [11]. In fact, the vascular perturbation contributing to hypertension leads to the activation of endothelial cells (EC) and the release of mediators promoting inflammation at level of different organs/cells (e.g., neuroinflammation), in the context of the mosaic theory of hypertension [12]. The interaction between the various players is too complex to be studied with current preclinical models, both in vitro and in vivo, and new advanced models are necessary to more deeply explore the underlying pathogenic mechanisms and to test new potentially therapeutic molecules.

Recently, our group developed an advanced fluidic multicompartmental model of in vitro hypertension able to reproduce some features of vascular function, like response to shear stress, and to simulate hemodynamic conditions by controlling flow and adjusting pressure parameters [13]. This innovative system can be used to biologically characterize and evaluate the potential therapeutic effects of novel chemical scaffolds [14] in hypertension.

**Figure 1:**
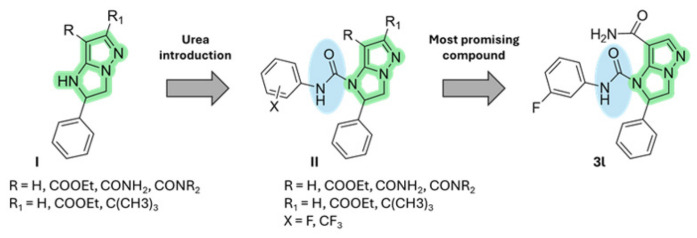
Molecular structure of imidazo-pyrazoles **I** and imidazo-pyrazolyl ureas (IPUs) **II** and IPU **3l**.

Effective chemical scaffolds are typically stable, versatile, and synthetically accessible, with inherent affinity for specific biological targets. Indeed, aromatic and heterocyclic systems containing heteroatoms (e.g., N, O, S) are among the most widely employed scaffolds in medicinal chemistry [15,16,17]. Heterocyclic nitrogen-containing scaffolds such as pyrazoles, imidazoles, and fused imidazo-pyrazoles are widely recognized for their biological activity across different therapeutic areas [18,19,20,21,22,23,24,25,26,27,28,29,30]. Building on this evidence, we recently developed a library of imidazo-pyrazole derivatives (compounds **I**, Figure 1), differently functionalized at positions C6 and C7, with the aim of exploring their structure–activity relationships (SARs) and identifying compounds with potential pharmacological relevance [14,31]. Among these, a subset of compounds (compounds **II**, Figure 1) was further modified by the introduction on N1 position of a urea moiety, a functional group capable of acting both as hydrogen bond donor and acceptor, thus potentially enhancing interactions with biological targets and improving aqueous solubility. Importantly, selected imidazo-pyrazolyl ureas (IPUs) demonstrated a marked ability to modulate inflammatory responses. In particular, these compounds potently inhibited polymorphonuclear neutrophil (PMN) chemotaxis (induced by both interleukin (IL)-8 and *N*-formyl-methionyl-leucil-phenylalanine (fMLP)), with IC_50_ values in the pico-nanomolar range, highlighting their strong anti-inflammatory potential [14]. Mechanistic studies further revealed that IPUs can modulate key signaling pathways involved in inflammation, including p38MAPK, ERK, PKC, and Akt kinases, which are also implicated in endothelial dysfunction and vascular inflammatory processes [14,32]. Among the tested compounds, IPU **3l** (Figure 1) showed the most pronounced inhibitory activity on p38MAPK, ERK, and PKC phosphorylation in PMNs, without impairing their bactericidal function, suggesting a selective anti-inflammatory profile [14,33]. Notably, no antiangiogenic or antitumoral effects were observed, supporting a mechanism of action primarily related to inflammation rather than cytotoxicity [34].

Since endothelial inflammation is a key contributor to the development and progression of hypertension, the ability of IPU **3l** to modulate inflammatory signaling pathways suggests a potential role for this compound in vascular protection. However, its effects on endothelial cells have not yet been investigated. To address this gap, we employed our previously developed in vitro model of hypertension, in which human umbilical vein endothelial cells (HUVECs) are exposed to ANGII and/or mechanical stress (Live-Pa), mimicking key chemical and hemodynamic stimuli associated with hypertensive conditions [13]. This model enables the evaluation of inflammatory mediators and signaling pathways involved in endothelial dysfunction.

Based on these considerations, we hypothesized that IPU **3l**, selected as the optimal IPU candidate, may exert protective effects in hypertension by modulating endothelial inflammatory pathways. Accordingly, the aim of this study was to investigate the effects of IPU **3l** in our in vitro hypertension model. Following the in silico evaluation of PK and PD properties, drug-likeness, and predicted oral toxicity, and after confirming the safety profile of IPU **3l** in HUVECs, we investigated its potential to modulate key pathways involved in endothelial inflammation and hypertension. Specifically, we focused on NF-κB and p38 MAPK signaling, as well as on the release of IL-6, IL-8, and endothelin-1 (ET-1), which are well-established mediators of vascular inflammation and endothelial dysfunction. These endpoints were selected to directly assess whether IPU **3l** can interfere with inflammatory mechanisms relevant to the development of hypertension in our in vitro experimental model.

## 2. Results

### 2.1. Pharmacokinetic Properties, Drug-Likeness and Toxicity Prediction

In the rational design of new compounds, in silico analysis represents a fundamental preliminary step to predict the PK properties and drug-likeness of the designed molecules; in this regard, the Swiss ADME software platform (http://swissadme.ch), developed by the Swiss Institute of Bioinformatics, was used to evaluate the pharmaceutical relevance of IPU **3l**, its PKs properties, and their drug-likeness [35].

This investigation provides a detailed analysis based on validated computational models, including molecular weight, the number of donor and acceptor hydrogen bonds (which influence solubility and permeability), the number of rotatable bonds (which is correlated with molecular flexibility), the logP (an indicator of lipophilicity and useful parameter for estimating the compound ability to cross cell membranes), and the TPSA (Topological Polar Surface Area) parameter. The latter represents the sum of the partial surfaces of the molecule polarized atoms, a crucial value for predicting cellular permeability, including CNS penetration and intestinal absorption [35]. In particular, a TPSA lower than 140 Å^2^ is believed to be favorable for oral absorption, while values lower than 90 Å^2^ are indicative of good blood–brain permeability. Swiss ADME also facilitates predicting drug-likeness information according to different rules (Lipinski, Ghose, Veber, Egan, Muegge) [36,37] and estimating whether a molecule could be a substrate of P-glycoprotein (P-gp), an efflux protein involved in reducing the bioavailability of various drugs, or an inhibitor of the main CYP isoenzymes. The number of violations of the Lipinski rules [36] for each synthesized molecule is also indicated.

The results of these investigations on IPU **3l** are reported in Table S1 and Figure S1 (Supplementary Materials). Specifically, the number of rotatable bonds is 5, the number of H-bond acceptors is 4, and the number of H-bond donors is 2, whereas TPSA is 93.25 Å^2^. IPU **3l** is predicted to have a good aqueous solubility (2.31 × 10^−1^ mg/mL, ESOL method), a good ability to permeate membrane cells (LogP values 1.52) and to permeate to the gastro-intestinal (GI) tract. On the contrary, the compound seems not able to permeate the blood–brain barrier (BBB) and to inhibit different CYP enzymes (1A2, 2C19, 2C9, 2D6, 3A4). In addition, no violations of the Lipinski rules were detected, and neither were any pan assay interference compound (PAINS) alerts found. Finally, IPU **3l** is predicted to be a PgP substrate.

The oral toxicity profile of IPU **3l** was predicted using the ProTox webserver [38] a virtual lab for the prediction of toxicities of small molecules. In detail, ProTox 3.0 combines several advanced techniques to predict how toxic a chemical might be to humans or the environment, covering a wide range of potential effects. More specifically, the program possesses as main key features: (a) “Molecular Similarity” analyzes how similar a molecule is to compounds known to be toxic; (b) “Fragment Propensities” examines the structural fragments of the molecule that could contribute to toxicity; (c) “Most Frequent Features” identifies the most common chemical features associated with toxic effects; (d) “Fragment Similarity-based CLUSTER Cross-Validation” is a machine learning approach to cluster data and improve predictions. The software uses 61 models to predict various toxicity endpoints, including: acute toxicity (to predict immediate and severe effects), specific organ toxicity, toxicological endpoints (to predict specific adverse effects), molecular initiating events (to evaluate initial interactions that can lead to toxicity), metabolism, adverse outcomes (to predict long-term adverse effects as carcinogenicity), and finally toxicity targets to identify proteins or biological pathways probably involved in toxicity.

According to the simulation, IPU **3l** seems to belong to class 4 toxicity (predicted LD_50_ value is 1000 mg/kg). Based on this analysis IPU **3l** is found to be free of hepatotoxicity, cardiotoxicity, immunotoxicity, cytotoxicity and nutritional toxicity; on the contrary some respiratory toxicity and neurotoxicity have been predicted (Figure S1, Supplementary Materials) [39].

Lastly, no toxicity target pharmacophores have been detected (Novartis off-targets, Adenosine A2a receptor, Adrenergic beta 2 receptor, Androgen receptor, Amine oxidase A, Corticotropin-releasing hormone receptor 1, Dopamine D3 receptor, Estrogen receptor 1, Estrogen receptor 2, Glucocorticoid receptor, Histamine H1 receptor, Nuclear receptor subfamily 1 group I member 2, Opiod receptor kappa 1, Progesterone receptor, Phosphodiesterase 4D, Prostaglandin G/H synthase 1).

Collectively, in silico predictions have shown that IPU **3l** exhibits promising PK characteristics, including adequate molecular weight, solubility and chemical stability consistent with a potential drug, good drug-like properties (e.g., logP, number of H-bond donors/acceptors) with a structure optimized for interactions with biological targets (e.g., enzymes, receptors), good intestinal absorption and tissue distribution, low acute and chronic toxicity, with no warning signals for mutagenicity, carcinogenicity or teratogenicity, and an adequate safety profile.

### 2.2. Cytotoxicity and Metabolic Activity of IPU ***3l***

**Figure 2:**
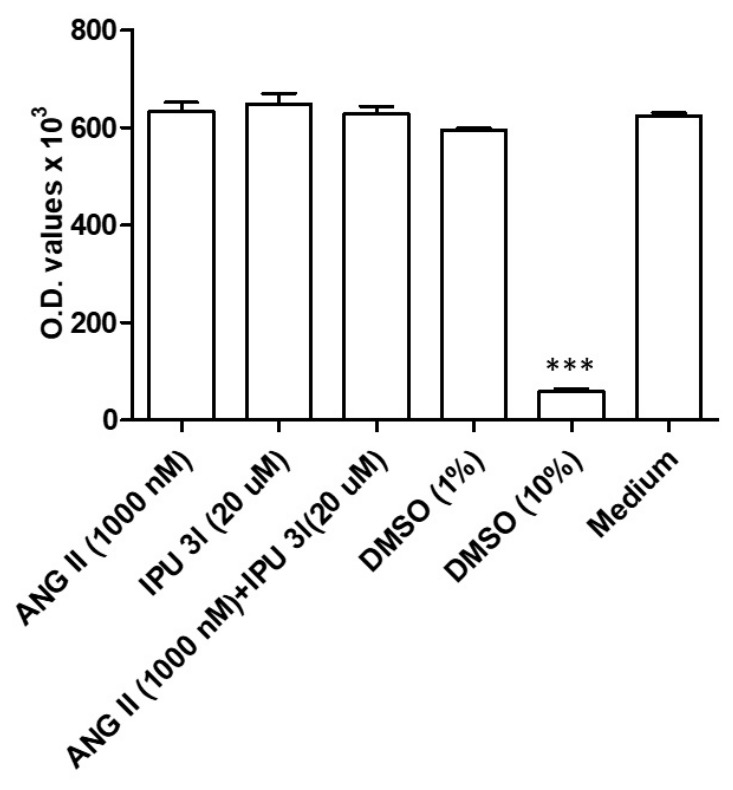
MTT assay. Cell viability and proliferative activity were evaluated by MTT assay in different experimental conditions. The intensity of the reaction is proportional to cell viability. Values are expressed as optical density (OD values × 10^3^), mean of three different experiments performed in different days with cells from different batches. Histograms represent mean + standard error of the mean (SEM). *** *p* < 0.001 versus lethal concentration of DMSO (10%).

To rule out the potential toxic effects of IPU **3l** alone or in combination with ANGII, the physical and functional integrity of HUVEC were evaluated by MTT assay. The compound did not affect cell viability and proliferative activity in all the experimental conditions. ANGII alone and DMSO as drug vehicle (1%) gave same results, at variance with DMSO at lethal concentration (10%), used as control of assay efficiency (Figure 2).

### 2.3. Modulatory Effects of IPU ***3l*** on Selected Signaling Pathways in HUVECs Grown in Our Multicompartmental Fluidic System

#### 2.3.1. NFκB and p38MAPK Phosphorylation Levels in Static Culture Conditions

HUVEC seeded in the LB1 bioreactor without flow were incubated with the positive control of hypertension ANGII (1000 nM, 24 h), IPU **3l** (20 µM, 20 min) alone or in combination with AngII, or culture medium.

**Figure 3:**
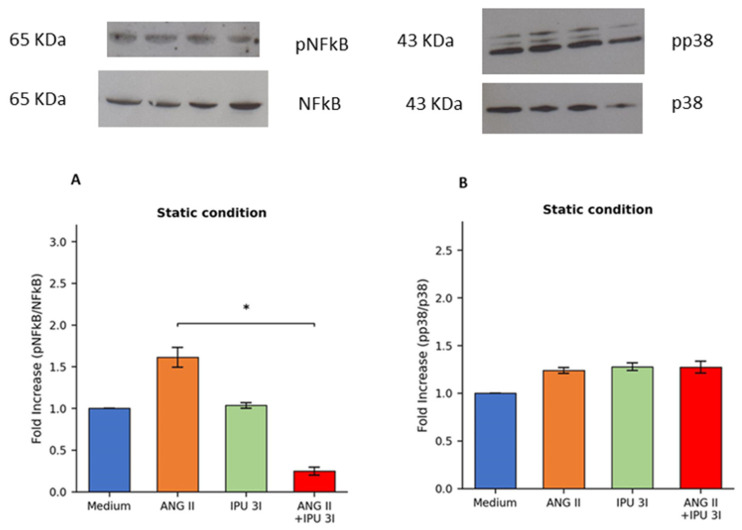
NF-κB (**A**) and p38MAPK (**B**) activation in HUVECs seeded in LB1 in static conditions. HUVECs were incubated with ANGII (1000 nM) for 24 h or IPU **3l** (20 μM) alone for 20 min or in combination with ANGII for the last 20 min or medium. Results are expressed as the ratio of phosphorylated to non-phosphorylated form, normalized to the control (medium). Histograms represent mean ± standard error of the mean (SEM) of three independent experiments (*n* = 3 per group). pN-FκB: phosphorylated NF-κB; pp38MAPK: phosphorylated p38MAPK. Statistical analysis: Kruskal–Wallis test followed by Dunn’s post hoc test with Benjamini–Hochberg FDR correction. * q < 0.05. Western blot images are representative of a single experiment.

The combination of ANGII and IPU **3l** resulted in an important reduction in NF-κB (Figure 3A) but not p38MAPK phosphorylation compared to ANGII alone (Figure 3B). (ANGII vs. combo q = 0.013 for pNF-κB).

#### 2.3.2. NF-κB and p38MAPK Phosphorylation Levels in Dynamic Conditions

Parallel experiments were performed in LB1 under controlled flow (100 µL/min) in the presence of ANGII (1000 nM, 24 h), IPU **3l** (20 µM, 20 min) alone or in combination with ANGII, or culture medium.

**Figure 4:**
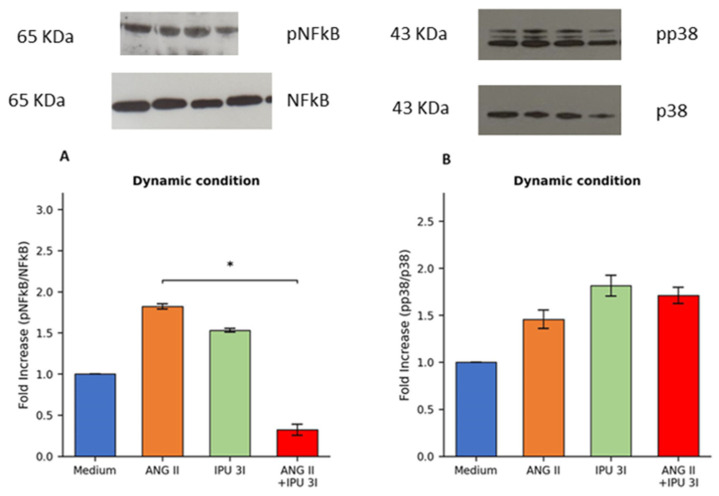
NF-κB (**A**) and p38MAPK (**B**) activation in HUVECs seeded in LB1 in dynamic conditions. HUVECs were incubated with ANGII (1000 nM) for 24 h or IPU **3l** (20 μM) alone for 20 min or in combination with ANGII for the last 20 min or medium. Results are expressed as the ratio of phosphorylated to non-phosphorylated form, normalized to the control (medium). Histograms represent mean ± SEM of three independent experiments (n = 3 per group). pNF-κB: phosphorylated NF-κB; pp38MAPK: phosphorylated p38MAPK. Statistical analysis: Kruskal–Wallis test followed by Dunn’s post hoc test with Benjamini–Hochberg FDR correction. * q < 0.05. Western blot images are representative of a single experiment.

Levels of NF-κB phosphorylation, when treated with **3l,** severely decreased (ANGII vs. combo q = 0.013) (Figure 4A), whereas p38MAPK signaling appeared slightly, but non-significant, enhanced (Figure 4B).

#### 2.3.3. NF-κB and p38MAPK Phosphorylation Levels in Our Dynamic Modular System Implemented with Live-PA Device

**Figure 5:**
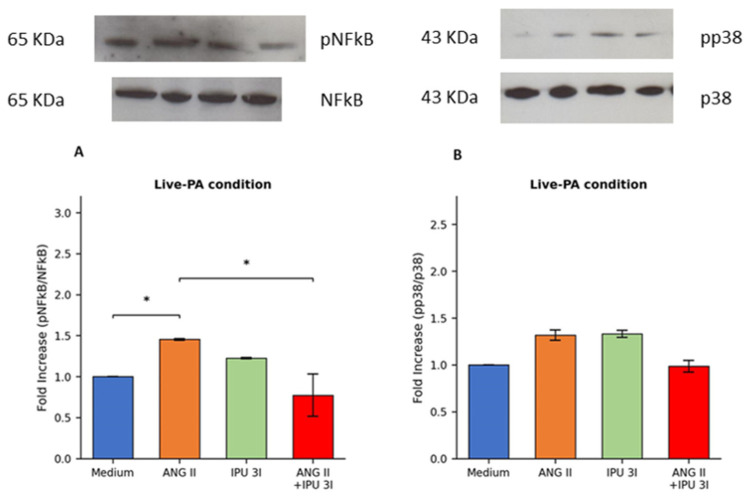
NF-κB (**A**) and p38MAPK (**B**) activation in HUVECs seeded in LB1 in dynamic conditions in the presence of Live-PA device as mechanical stimulus. HUVECs were incubated with ANGII (1000 nM) for 24 h or IPU **3l** (20 μM) alone for 20 min or in combination with ANGII for the last 20 min or medium. Results are expressed as the ratio of phosphorylated to non-phosphorylated form, normalized to the control (medium). Histograms represent mean ± SEM of three independent experiments (n = 3 per group). pNF-κB: phosphorylated NF-κB; pp38MAPK: phosphorylated p38MAPK. Statistical analysis: Kruskal–Wallis test followed by Dunn’s post hoc test with Benjamini–Hochberg FDR correction. * q < 0.05. Western blot images are representative of a single experiment.

Live-PA device was applied to HUVEC cultured under flow (100 µL/min), as a mechanical stimulus, for the last 2 h of culture in the presence of ANGII (1000 nM) or IPU **3l** (20 μM, 20 min) alone or in combination with ANGII, or medium alone. The IPU **3l** alone upregulated both signaling pathways. The combination of ANGII and IPU **3l** significantly reduced NF-κB activation in comparison with ANGII alone (Figure 5A); a similar downward trend was observed for p38MAPK activation, although it did not reach statistical significance (Figure 5B).

### 2.4. Effects of IPU ***3l*** on Secretion of Inflammatory Mediators by HUVEC Cultured in Our Multicompartmental Fluidic System

#### 2.4.1. Measurement of Cytokine/Chemokine Secretion in Static Culture Conditions

**Figure 6:**
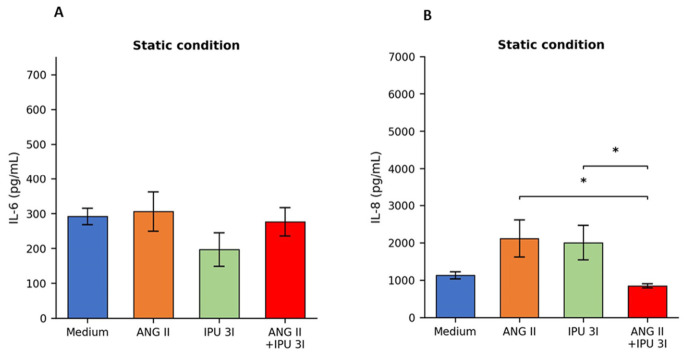
IL-6 (**A**) and IL-8 (**B**) secretion in HUVEC supernatants in static conditions. Endothelial cells were incubated with ANGII (1000 nM) for 24 h or IPU **3l** (20 μM) alone for 20 min or IPU **3l** with ANGII for the last 20 min. Histograms represent mean ± SEM. Sample sizes per group: IL-6: medium n = 13, ANGII n = 7, IPU **3l** n = 3, ANGII + IPU **3l** n = 7; IL-8: medium n = 11, ANGII n = 4, IPU **3l** n = 5, ANGII + IPU **3l** n = 3. Statistical analysis: Kruskal–Wallis test followed by Dunn’s post hoc test with Benjamini–Hochberg FDR correction. * q < 0.05.

In the absence of flow, ANGII (1000 nM) 24 h incubation induced a trend toward increased IL-8 secretion (q = 0.059) in HUVEC supernatants. The exposure of the cells to IPU **3l** (20 μM) for 20 min did not significantly change IL-6 secretion, with no significant change in IL-8 levels compared to the medium. Of note, the addition of IPU **3l** to ANGII for the last 20 min of culture did not significantly affect IL-6 secretion (Figure 6A), while it caused a statistically significant downregulation of IL-8 levels versus ANGII alone, and versus IPU **3l** alone (Figure 6B).

#### 2.4.2. Quantification of IL-6 and Il-8 Secretion Levels in Dynamic Conditions

HUVEC cultured under flow and incubated with ANGII (1000 nM, 24 h) showed an increase in both IL-6 and IL-8 secretion, reaching statistical significance for IL-8 only (*p* < 0.01).

**Figure 7:**
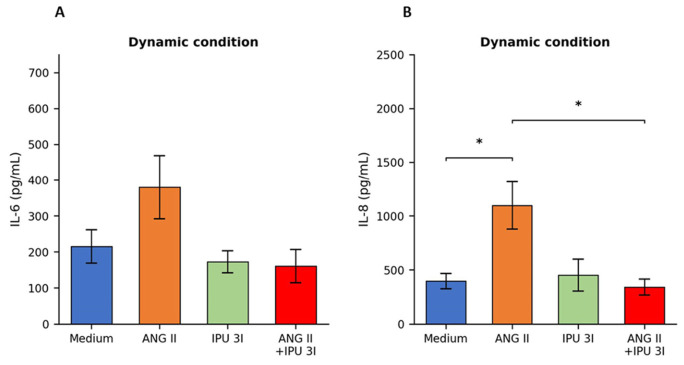
IL-6 (**A**) and IL-8 (**B**) secretion in HUVEC supernatants in dynamic conditions. Endothelial cells were incubated with ANGII (1000 nM) for 24 h or IPU **3l** (20 μM) alone for 20 min or IPU **3l** with ANGII for the last 20 min. Histograms represent mean ± SEM. Sample sizes per group: IL-6: medium n = 5, ANGII n = 4, IPU **3l** n = 4, ANGII + IPU **3l** n = 4; IL-8: medium n = 8, ANGII n = 4, IPU **3l** n = 4, ANGII + IPU **3l** n = 4. Statistical analysis: Kruskal–Wallis test followed by Dunn’s post hoc test with Benjamini–Hochberg FDR correction. * q < 0.05.

The addition of IPU **3l** to ANGII-stimulated HUVEC downregulated IL-6 (q = ns) and even more IL-8 (q = 0.039), as compared to ANGII alone, bringing back mediator to the medium basal levels (Figure 7).

#### 2.4.3. Measurement of IL-6 and IL-8 Levels in Dynamic Modular System Implemented with Live-PA Device

**Figure 8:**
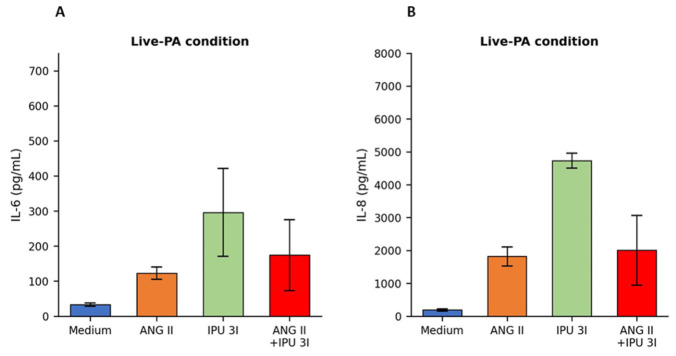
IL-6 (**A**) and IL-8 (**B**) secretion in HUVEC supernatants in the presence of the mechanical stimulus Live-Pa. Endothelial cells were incubated with ANGII (1000 nM) for 24 h or IPU **3l** (20 μM) alone for 20 min or IPU **3l** with ANGII for the last 20 min in the presence of Live-Pa applied in the final 2 h of ANGII stimulation. Histograms represent mean ± SEM. Sample sizes per group: IL-6: medium n = 2, ANGII n = 2, IPU **3l** n = 3, ANGII + IPU **3l** n = 2; IL-8: medium n = 2, ANGII n = 2, IPU **3l** n = 2, ANGII + IPU **3l** n = 2. Statistical analysis: Kruskal–Wallis test followed by Dunn’s post hoc test with Benjamini–Hochberg FDR correction.

The dynamic incubation of HUVEC with ANGII (1000 nM, 24 h) applying the Live-Pa for the last two hours was associated with numerically increased IL-6 and IL-8 secretion in cell supernatants, although this did not reach statistical significance after correction for multiple comparisons. Similarly, IPU **3l** alone (20 μM, 20 min) induced a numerical increase in IL-6 and IL-8 in comparison with the Live-Pa alone, without reaching statistical significance. The co-presence of ANGII and IPU **3l** numerically enhanced both IL-6 and IL-8 levels, as compared to either Live-Pa or ANGII alone, although these differences were not statistically significant (Figure 8).

#### 2.4.4. Quantification of ET-1 Release by HUVEC Cultured in Static Environment or Under Flow Without or with the Live-Pa Device

**Figure 9:**
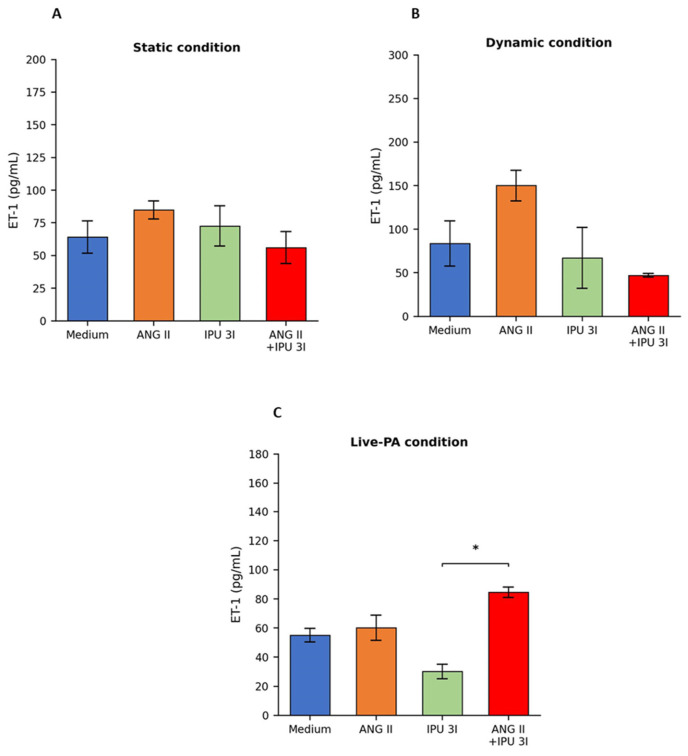
ET-1 secretion in HUVEC stimulated with ANGII (1000 nM) for 24 h or IPU **3l** (20 μM) alone for 20 min or together with ANGII for the last 20 min static (**A**) or dynamic conditions (**B**) or in combination with Live-Pa (**C**) for the last 2 h of ANGII stimulation. Histograms represent mean ± SEM. Sample sizes per group: static: medium n = 3, ANGII n = 3, IPU **3l** n = 2, ANGII + IPU **3l** n = 3; dynamic: medium n = 3, ANGII n = 3, IPU **3l** n = 2, ANGII + IPU **3l** n = 2; Live-Pa: medium n = 3, ANGII n = 3, IPU **3l** n = 2, ANGII + IPU **3l** n = 2. Statistical analysis: Kruskal–Wallis test followed by Dunn’s post hoc test with Benjamini–Hochberg FDR correction. * q < 0.05.

The incubation with IPU **3l** alone (20 μM) induced a mild, non-significant increase in the vasoconstrictor protein in static condition (Figure 9A). Under dynamic conditions, no pairwise comparison reached statistical significance after correction for multiple comparisons (Figure 9B). When the Live-Pa was applied to the dynamic system, ANGII-exposed HUVEC secreted a numerically increased amount of ET-1, although group differences did not reach significance for this comparison; notably, the co-presence of ANGII and IPU **3l** significantly enhanced ET-1 secretion compared to IPU **3l** alone under Live-Pa (q < 0.05, Figure 9C).

## 3. Discussion

This study confirms the usefulness of our previously developed dynamic in vitro model of hypertension as a reliable platform to investigate the pharmacological potential of novel compounds [13]. Using this platform, we evaluated the activity of IPU **3l** in a hypertensive environment, highlighting a context-dependent modulation of endothelial inflammatory responses.

Hypertension is a multifactorial disease in which inflammation plays a central role, with the endothelium representing one of the earliest targets of vascular damage [6,11,40,41]. Accordingly, using our in vitro model of hemodynamic stress we observed that HUVECs directly exposed to both chemical (ANGII) and mechanical (Live-Pa-induced pressure) stimuli actively contribute to vascular inflammation, through the release of inflammatory and vasoconstriction mediators, reproducing key features of the hypertensive microenvironment [13].

The development of new anti-hypertensive agents remains a main goal for the improvement of the management of hypertensive patients. In this context, IPU **3l** was selected based on its previously reported anti-inflammatory activity on PMNs [14,33] and its PD, PK and drug-like properties, together with the absence of cytotoxic effects in HUVECs (Figure 2), supported its further evaluation in our experimental model.

Under static conditions, IPU **3l** attenuated ANGII-induced NF-κB activation (combo vs. ANGII, q = 0.0125; Figure 3), without significantly affecting p38 MAPK phosphorylation, and the combination with ANGII was associated with a significant reduction in IL-8 levels compared to ANGII alone (q = 0.0355; Figure 6B), with no significant change in ET-1 levels (Figure 9A).

Under flow conditions reproducing physiological shear stress, IPU **3l** confirmed the reduction in ANGII-induced NF-κB activation and IL-8 levels (Figure 4A and Figure 7B); the reduction in ET-1 levels under these conditions (Figure 9B) did not reach statistical significance after correction for multiple comparisons.

In contrast, when mechanical stress was applied alone through the Live-Pa system, IPU **3l** was associated with a non-significant numerical decrease in ET-1 secretion compared to Medium and ANGII (Figure 9C), while no significant differences were detected among groups for IL-6 or IL-8 (Figure 8).

Finally, when chemical and mechanical stimuli were combined (ANGII + Live-Pa), IPU **3l** significantly reduced NF-κB phosphorylation (Figure 5A), confirming this as the most consistent pharmacological effect of the compound across all experimental conditions; p38MAPK phosphorylation showed a similar downward trend, but did not reach statistical significance (Figure 5B). This effect on NF-κB was not consistently associated with a decrease in downstream inflammatory mediators.

Notably, the behavior of ET-1 under Live-Pa conditions was particularly informative: IPU **3l** was associated with numerically lower ET-1 levels when applied alone, whereas the combination of IPU **3l** and ANGII was associated with significantly higher ET-1 levels compared with IPU **3l** alone. This observation illustrates the value of a system capable of separating chemical and mechanical hypertensive stimuli: a conventional static culture or an in vivo model, where these two stressors are inherently superimposed, would not have been able to reveal this condition-specific divergence. This observation has important implications for the design of subsequent drug-testing studies involving IPU **3l**, highlighting the need to explicitly consider the combined mechanical and chemical components of the hypertensive stimulus rather than assuming that they are interchangeable. Taken together, these findings suggest that IPU **3l** acts in a pathway-specific rather than broadly anti-inflammatory manner: its effect is robust and consistent on NF-κB, with corresponding effects on IL-8, but does not extend to comprehensive protection across all vascular mediators when chemical and mechanical hypertensive stimuli are combined.

Importantly, our dynamic model, by allowing discrimination between the individual and combined effects of chemical and mechanical hypertensive stimuli, a resolution unattainable in conventional in vitro or in vivo experimental models, enabled a level of pharmacological detail that directly informs the design of future mechanistic studies.

The different behavior observed between PMNs and HUVECs [31,33] further suggests a cell-type-specific mechanism of action, likely reflecting differences in intracellular signaling, receptor expression, metabolic processing and biological functions [11].

In summary, the ability of this setting to discriminate between IPU **3l**’s pharmaco-logical response to ANGII and to mechanical stress provides two very important strengths. First, by showing that the molecule exerted its protective properties only under ANGII stimulus, it suggests a possible mechanistic hypothesis and an experimental roadmap for subsequent studies. Second, it demonstrates condition-specific loss of efficacy that would remain invisible in conventional static in vitro models or in vivo systems, where biochemical and mechanical stimuli cannot be decoupled.

## 4. Material and Methods

### 4.1. Synthesis of IPU ***3l***

Pure IPU **3l** (>95% purity, as determined by elemental analyzer EA 1110), was obtained in sufficient amounts to perform the planned biological tests as previously reported [31].

### 4.2. In Silico Prediction of ADMET Properties

The pharmaceutical relevance of IPU **3l**, its PK s properties, and their drug-likeness, were calculated using SwissADME [35], whereas its toxicity profile was predicted using the ProTox webserver [38].

### 4.3. Cell Culture Model

An innovative modular system developed by IVTech (IVTech Srl, Ospedaletto, PI, Italy) for 2D and 3D cell culture, in static or dynamic milieu, was used in this study. The device is composed of a bioreactor, Live Box (LB)1, equipped by a removable glass slide where endothelial cells might build a monolayer, and by a tube inlet and outlet for the passage of the culture medium. A peristaltic pump (LiveFlow) with an adjustable flow rate ranging from 100 to 450 μL/min was connected to both LB1 and a medium reservoir. This pump was able to maintain a closed-loop circulation with a horizontal flow directly to the endothelial monolayer and to reproduce blood vessel circulation without extravasation.

The shear stress (τ), applied to endothelial monolayer in this experimental condition (flow 100 µL/min) and calculated by a general formula τ=−µx(dv/dz) is 6 × 10^−10^ Pa (Pascal) (µ viscosity of the fluid and dν/d*z* represented the velocity gradient across the channel’s cross-section). The calculation was performed automatically by the numerical simulation software application Simflow (Version 5.1), which generated a colorimetric map of shear stress distribution across the LB1 slide surface.

To simulate hypertensive conditions, the system was integrated with a Live-Pa pressure modulator, which used a motorized piston to constrict the outlet tubing, thereby increasing hydrodynamic pressure. The Live-Pa device can operate within a pressure range of 101.3 to 202.6 Pascal (0.75 to 1.52 mmHg), corresponding to 50%, 100%, and 200% incremental increases over the basal system pressure of 101.3 Pa (0.75 mmHg).

To better reproduce human hypertension disease a 50% pressure increase (from 101.3 to 151.1 Pa; 0.75 to 1.13 mmHg) was selected as the most appropriate setting to model the hemodynamic perturbation characteristic of hypertension. As we were operating in a close-loop system, LiveFlow imposed a constant flow, except the section of the tube where Live-PA was located.

It is important to note that the crucial parameter in this model is the ratio between applied overpressure and basal system pressure, rather than the absolute values themselves. As this is a scaled millifluidic model, there is no direct 1:1 correspondence between the pressures used in the device and in vivo arterial pressures. The biological validity of this approach is supported by the fact that the cellular responses obtained, specifically the activation of NF-κB, p38MAPK, and IL-8, are consistent with those observed in established in vivo hypertension models, as detailed in the companion validation paper [13].

### 4.4. Cell Culture Treatment

Human umbilical vein endothelial cells were purchased from Promocell (Cat.No. C-12203, Heidelberg, Germany) and cultured in the provided Endothelial Cell Growth Medium Ready-to-use (Promocell) in a humidified incubator at 37 °C with 5% CO_2_. Upon reaching confluence, cells were passaged using Detach kit-30 (Promocell), and 20,000 cells per 96-well plate and 200,000 passaged in LB1 were seeded in complete medium and cultured in static condition for 24 h to reach a monolayer.

Cells were then incubated with the pharmacological positive control of hypertension (ANGII; 1000 nM for 24 h [42]); IPU **3l** (20 µM) for 20 min or ANGII (1000 nM) for 24 h in combination with IPU **3l** (20 µM) for the last 20 min or medium alone, in static and dynamic conditions. Parallel experiments were carried out for the last 2 h in the presence of Live-PA at 50% pressure increase as mechanical stimuli.

HUVEC morphology and monolayer integrity were observed using inverted microscopy (Wilovert, Wetzlar, Germany) across all the experimental conditions.

### 4.5. Cytotoxic Assay

To evaluate cell viability, MTT assay was performed in 96-well plate on HUVEC monolayer.

Cells were treated with AngII (1000 nM) for 24 h; IPU **3l** (20 µM) for 20 min or ANGII for 24 h in combination with IPU **3l** for the last 20 min; DMSO (1%) as vehicle for 20 min; DMSO (10%) at lethal concentration or medium alone were also included as positive and negative controls, respectively. This method is defined as a colorimetric assay that measures cell metabolic activity to evaluate the number of viable cells by quantifying the conversion of the yellow tetrazolium salt MTT into insoluble purple formazan crystals through a reduction reaction in living cells. The concentration of formazan is measured using a spectrophotometer, correlating higher cell proliferation with increased MTT conversion.

### 4.6. Western Blot Analysis

Total proteins from HUVECs seeded in LB1 and exposed to the experimental conditions described above were purified using RIPA Lysis Buffer (Cell Signaling Technology, Danvers, MA, USA) supplemented with 1% Protease and Phosphatase Inhibitor Cocktail (Sigma-Aldrich, Burlington, MA, USA). Protein concentrations were calculated using the BCA Protein Assay Kit (ThermoFisher Scientific, Waltham, MA USA) and separated by NuPAGE Bis-Tris 4–12% SDS-polyacrylamide gel electrophoresis using pre-cast gels (ThermoFisher Scientific) and then transferred to nitrocellulose membranes using iBlot 3 Transfer Stacks (ThermoFisher Scientific).

Membranes were blocked for 2 h at room temperature in PBS containing 0.05% Tween 20 (PT; Cell Signaling Technology) and 5% non-fat dry milk (Santa Cruz Biotechnology, Dallas, TX, USA), then incubated overnight at 4 °C with primary antibodies against human NF-κB or phosphorylated NF-κB (pNF-κB) or p38MAPK or phosphorylated p38MAPK (pp38MAPK; Cell Signaling Technology). After three washes, HRP-conjugated secondary antibodies (Cell Signaling Technology, Danvers, MA, USA) were added for 1 h at room temperature. Protein bands were visualized using enhanced chemiluminescence (ECL; Westar Supernova, Cyanagen, Bologna, Italy) and detected on radiographic films (Kodak, Rochester, NY, USA). Intensity of the bands was analyzed and quantified using ImageJ software (ImageJ 1.53e, LI-COR Biosciences, Lincoln, NE, USA). Results were expressed as the ratio of phosphorylated to non-phosphorylated protein forms, normalized to the respective controls.

### 4.7. Measurement of Mediator Levels in Cell Supernatants

IL-6 and IL-8 were measured using the automated microfluidic analyzer ELLA (Bio-Techne, Minneapolis, MN, USA), while ET-1 was quantified by commercial ELISA kit (Bio-Techne, Minneapolis, MN, USA), in supernatants collected at the end of each experiment, according to the manufacturer’s instructions, as previously reported [13]. The concentrations of each analyte (pg/mL, mean of three reading) were obtained from the specific calibration curve using the system’s software. Minimum detectable level for IL-6 was 0.7 pg/mL (range 0.7–2.652 pg/mL); for IL-8 0.08 pg/mL (range 0.08–1.8 pg/mL) and for ET-1 0.031 (range 0.031–0.207 pg/mL).

### 4.8. Statistical Analysis

For the cytotoxicity assay, continuous variables are expressed as mean ± standard error of the mean (SEM); a one-way ANOVA followed by Tukey’s post hoc test was used to evaluate differences between groups.

For all other experiments (Western blot and ELLA), group differences were assessed using the Kruskal–Wallis test; when a significant overall effect was detected, pairwise comparisons were performed using Dunn’s post hoc test. To control for multiple comparisons across the four experimental groups (Medium, ANGII, IPU **3l**, ANGII + IPU **3l**), q-values were corrected using the Benjamini–Hochberg False Discovery Rate (BH-FDR) procedure; corrected *p*-values are reported as q-values. Data are expressed as mean ± SEM. All the analyses were performed with GraphPad Prism 5.01. Statistical significance was set at the 0.05 level.

## 5. Conclusions

This study represents a proof-of-concept phase of our stepwise research program; it employed a recently developed model of hypertension partially reproducing the blood flow in vivo [13], to provide encouraging evidence of the pharmacological potential of the IPU **3l** in the context of hypertension. The compound showed a favorable in silico safety and drug-like profile and did not exert cytotoxic effects on HUVECs. Mechanistically, IPU **3l** consistently reduced ANGII-induced NF-κB activation, a key pathway in vascular inflammation, across all three experimental conditions tested (static, dynamic, and in combination with Live-Pa), and reduced IL-8 secretion under static and dynamic conditions. A similar downward trend on p38MAPK activation was observed when ANGII was combined with Live-Pa, although it did not reach statistical significance. Overall, despite context-dependent effects on other mediators such as ET-1, the consistent modulation of the NF-κB/IL-8 axis supports the potential of IPU **3l** as a candidate for further preclinical investigation.

Additionally, our findings suggest that the molecule may differentially modulate distinct components of hypertension, providing preliminary insights for future targeted therapeutic strategies. Importantly, the differential activity of IPU **3l** observed in different cellular settings, particularly in our dynamic system combined with mechanical stimulation, highlights the necessity of such advanced experimental systems, integrating both chemical and mechanical hypertensive stimuli, in the drug discovery process. Such an approach may improve the prediction of in vivo behavior of potential therapeutic compounds.

While this study provides valuable preliminary evidence of the protective effects of the tested molecule in a simulated hypertensive environment, some limitations must be acknowledged. First, although we observed a significant modulation of key inflammatory markers, our current experimental design was primarily observational. Consequently, while we can document the compound’s effects, we cannot yet provide definitive mechanistic insights or a complete mapping of the underlying molecular interactions.

Additional orthogonal assays would provide stronger validation of target engagement; however, the scope of the present study was focused on functional readouts in a dynamic in vitro model. A direct mechanistic validation on endothelial cells, including the use of selective pathway inhibitors (e.g., losartan for AT1R), as pharmacological comparators, and a reference antihypertensive or anti-inflammatory compounds as a positive pharmacological control is planned as a priority in the next phase of our research program, where they will serve both as mechanistic validation tools and as pharmacological comparators for novel compound screening.

The concentration and exposure conditions used in this study were selected based on previously published works on other IPUs structurally similar to **3l** which have been evaluated in HUVECs [32].

Furthermore, this study utilized a 2D monoculture model of HUVEC, which, while useful for isolating endothelial responses, lacks the complex paracrine crosstalk provided by other vascular cells or the immune system.

It should also be noted that the small number of independent biological replicates (n = 3 for Western blot experiments), while appropriate for an exploratory proof-of-concept study, limits the statistical power of the analyses and the generalizability of the findings. Larger confirmatory studies will be necessary to establish the robustness and magnitude of the observed effects.

In conclusion, two main messages can be conveyed: (i) the dynamic platform successfully reveals condition-specific pharmacological responses, enabling a level of discrimination between chemical and mechanical hypertensive stimuli that would remain obscured in standard static cultures or masked by the systemic complexity of in vivo models; (ii) IPU **3l** shows a robust and consistent inhibitory effect on the NF-κB/IL-8 axis in ANGII-stimulated HUVECs, while its effects on other vascular mediators, such as ET-1, are condition-dependent and do not extend uniformly across all experimental settings, particularly when chemical and mechanical hypertensive stimuli are combined. This pathway-specific profile, together with the platform’s ability to discriminate between distinct hypertensive stressors, provides a precise roadmap for future investigation with appropriate mechanistic tools.

## Data Availability

The original contributions presented in this study are included in the article/Supplementary Material. Further inquiries can be directed to the corresponding author. Samples of compound IPU **3l** are available from the authors. The datasets generated for this study can be found in the ZENODO repository DOI:10.5281/zenodo.19494683 (https://doi.org/10.5281/zenodo.19494683).

## Supplementary Materials

The following supporting information can be downloaded at: https://www.mdpi.com/article/10.3390/ph19071003/s1, Table S1. Predicted PK and drug-like properties of compounds; Figure S1. Radar plot calculated for IPU **3l**.

## Abbreviations

The following abbreviations are used in this manuscript:

AngII	Angiotensin II
BBB	Blood–brain barrier
EC	Endothelial cells
ET-1	Endothelin-1
fMLP	N-Formylmethionine-leucyl-phenylalanine
GI	Gastrointestinal
HUVEC	Human umbilical vein endothelial cells
IL-6	Interleukin-6
IL-8	Interleukin-8
IPU	Imidazo-pyrazolyl urea
LB-1	LiveBox-1
NF-κB	Nuclear Factor kappa-light-chain-enhancer of activated B cells
PAINS	Pan-Assay Interference Compounds
PD	Pharmacodynamic
P-gp	P-glycoprotein
PK	Pharmacokinetic
p38MAPK	p38 mitogen-activated protein kinases
PMNs	Polymorphonuclear neutrophils
SAR	Structure–activity relationship
SEM	Standard error of the mean
TPSA	Topological Polar Surface Area
