# Supplementary material for: Modulation of Endothelial Inflammatory Signature by an Imidazo-Pyrazolyl Urea Derivative in a Dynamic In Vitro Model of Hypertension
Source: Pharmaceuticals (Basel). 2026 Jun 28;19(7):1003. doi: 10.3390/ph19071003 (PMC13416312; doi:10.3390/ph19071003)

Supplementary Materials

Modulation of Endothelial Inflammatory Signature by an Imidazo-Pyrazolyl Urea Derivative in a Dynamic In Vitro Model of Hypertension

Matteo Lusardi [1,†], Caterina Bodio [2,†], Chiara Brullo [1], Gianfranco Parati [3,4], Pier Luigi Meroni [2], Maria Orietta Borghi [2,5], Elena Raschi [2,‡] and Laura Calvillo [3,*,‡]

[1] Department of Pharmacy, Section of Medicinal Chemistry, University of Genova, Viale Benedetto XV 3, 16132 Genova, Italy; matteo.lusardi@edu.unige.it (M.L.); chiara.brullo@unige.it (C.B.)
[2] Immunorheumatology Research Laboratory, Istituto Auxologico Italiano, IRCCS, Via Zucchi 18, 20095 Milan, Italy; c.bodio@auxologico.it (C.B.); pierluigi.meroni@unimi.it or p.meroni@auxologico.it (P.L.M.); maria.borghi@unimi.it or o.borghi@auxologico.it (M.O.B.); raschi@auxologico.it (E.R.)
[3] Department of Cardiology, Istituto Auxologico Italiano, IRCCS, Via Magnasco 2, 20149 Milan, Italy; gianfranco.parati@unimib.it
[4] Department of Medicine and Surgery, University of Milano-Bicocca, Piazza dell'Ateneo Nuovo 1, 20126 Milan, Italy
[5] Dipartimento di Scienze Cliniche e di Comunità, Dipartimento di Eccellenza 2023–2027, University of Milan, Via Festa del Perdono 7, 20122 Milan, Italy
* Correspondence: l.calvillo@auxologico.it
† These authors have contributed equally to this work and share first authorship.
‡ These authors have contributed equally to this work and share last authorship.

Table of contents

Table S1. Predicted pharmacokinetics and drug-like properties of IPU **3l**.

Figure S1. Radar plot calculated for IPU **3l**.

Table S1. Predicted pharmacokinetics and drug-like properties of compounds.

	**3l**
**Physicochemical Prop.**	
MW (g/mol)	365.36
Fraction Csp^3	0.11
Rotatable bonds	5
H-bond acceptors	4
H-bond donors	2
TPSA[a] ($Å^2$)	93.25
**Lipophilicity**	
LogP[b]	1.52
**Water solubility**	
Solubility (mg/mL)[c]	2.31 x 10^{-1} mg/mL
Solubility class	soluble
**Pharmacokinetics**	
GI absorption	hight
BBB permeant	no
Pgp substrate	yes
CYP1A2 inhibitor	no
CYP2C19 inhibitor	no
CYP2C9 inhibitor	no
CYP2D6 inhibitor	no
CYP3A4 inhibitor	no
**Drug-likeness**	
Lipinski violations	0
**Medicinal chemistry**	
PAINS alerts	0
Brenk alerts	0

[a] Topological Polar Surface Area. [b] Predicted according to XLOGP3 program. [c] Values predicted by ESOL method.

Figure S1. Radar plot calculated for IPU **3l**.

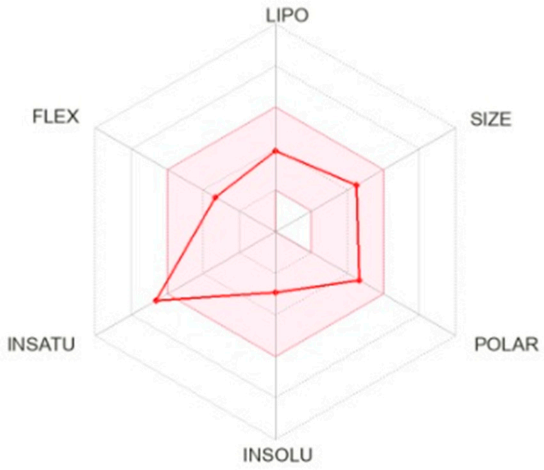

Supplement: Supplementary file 1 [file pharmaceuticals-19-01003-s001.zip › pharmaceuticals-4290651-supplementary - Copy.pdf]
